# The Spindle Matrix Protein, Chromator, Is a Novel Tubulin Binding Protein That Can Interact with Both Microtubules and Free Tubulin

**DOI:** 10.1371/journal.pone.0103855

**Published:** 2014-07-29

**Authors:** Changfu Yao, Chao Wang, Yeran Li, Yun Ding, Uttama Rath, Saheli Sengupta, Jack Girton, Kristen M. Johansen, Jørgen Johansen

**Affiliations:** Department of Biochemistry, Biophysics, and Molecular Biology, Iowa State University, Ames, Iowa, United States of America; Institut de Génétique et Développement de Rennes, France

## Abstract

The chromodomain protein, Chromator, is localized to chromosomes during interphase; however, during cell division together with other nuclear proteins Chromator redistributes to form a macro molecular spindle matrix complex that embeds the microtubule spindle apparatus. It has been demonstrated that the CTD of Chromator is sufficient for localization to the spindle matrix and that expression of this domain alone could partially rescue *Chro* mutant microtubule spindle defects. Furthermore, the presence of frayed and unstable microtubule spindles during mitosis after Chromator RNAi depletion in S2 cells indicated that Chromator may interact with microtubules. In this study using a variety of biochemical assays we have tested this hypothesis and show that Chromator not only has binding activity to microtubules with a Kd of 0.23 µM but also to free tubulin. Furthermore, we have mapped the interaction with microtubules to a relatively small stretch of 139 amino acids in the carboxy-terminal region of Chromator. This sequence is likely to contain a novel microtubule binding interface since database searches did not find any sequence matches with known microtubule binding motifs.

## Introduction

During cell division the entire nucleus undergoes a dramatic reorganization as the cell prepares to segregate its duplicated chromosomes. In *Drosophila* we have identified four nuclear proteins, Skeletor, Chromator, Megator, and EAST from two different nuclear compartments that interact with each other [1]–[4] and that redistribute during prophase to form a dynamic, gel-like spindle matrix that embeds the microtubule spindle apparatus, stretching from pole-to-pole [5]. This matrix forms prior to nuclear envelope breakdown and specific interactions between spindle matrix molecules are necessary for complex formation and cohesion [5]. When microtubules are depolymerized with colchicine just prior to metaphase the spindle matrix contracts and coalesces around the chromosomes suggesting that microtubules act as “struts” stretching the spindle matrix. For such a matrix to be stretched infers that components of the matrix physically be linked to microtubules and that changes to the shape and form of the matrix in turn are governed by microtubule dynamics [5]. Furthermore, in colchicine treated embryos free tubulin accumulates co-extensively with the spindle matrix proteins [5] suggesting that this enrichment is dependent on one or more proteins within the spindle matrix with tubulin binding activity.

A candidate spindle matrix protein for having tubulin binding activity is the chromodomain containing protein, Chromator, which during interphase is localized to interband regions of chromosomes [2]. Chromator can be divided into two main domains, an NH_2_-terminal domain (NTD) containing the chromodomain (ChD) and a COOH-terminal domain (CTD) containing a nuclear localization signal [2]. Recently, Yao et al. [6] provided evidence that the NTD of Chromator is responsible for correct targeting to chromatin, that it interacts with histone H1, and that the chromodomain is required for these interactions. Interestingly, the studies of Ding et al. [7] showed that the CTD of Chromator was sufficient for localization to the spindle matrix and that expression of this domain alone could partially rescue *Chro* mutant microtubule spindle defects. Furthermore, the presence of frayed and unstable microtubule spindles during anaphase after Chromator RNAi depletion in S2 cells indicated that Chromator may directly interact with microtubules [7]. Therefore, in this study we have explored this hypothesis by performing a variety of biochemical tubulin binding and interaction assays. The results show that a novel amino acid sequence in the CTD of Chromator has the capacity to bind both free and polymerized tubulin.

## Materials and Methods

### *Drosophila melanogaster* stocks and transgenic flies

Fly stocks were maintained according to standard protocols [8]. Transgenic flies expressing full-length, GFP-tagged Chromator under *GAL-4* promoter control have been previously characterized [5], [7]. Tubulin-mCherry (stock 25774) and a *tubulin-GAL-4* driver line (stock 7062) were obtained from the Bloomington *Drosophila* Stock Center, Indiana University, Bloomington, IN.

### Immunoblot analysis

Protein lysates were separated by SDS-PAGE and immunoblotted according to standard procedures [9]. For these experiments we used the Bio-Rad Mini PROTEAN III system, electroblotting to 0.2 µm nitrocellulose, and using anti-mouse, anti-goat or anti-rabbit HRP-conjugated secondary antibody (Bio-Rad) (1∶3000) for visualization of primary antibody. Primary antibodies used in this study included Chromator mAbs 6H11 and 12H9 [2], anti-GST mAb 8C7 [2], and mouse anti-tubulin (Sigma). Antibody labeling was visualized using chemiluminescent detection methods (SuperSignal West Pico Chemiluminescent Substrate or the SuperSignal kit from Pierce). The immunoblots were either digitized using a ChemiDoc-It TS2 Imager equipped with an epifluorescence attachment (UVP) or with a flatbed scanner (Epson Expression 1680).

### Overlay experiments

For the overlay experiments GST-tagged versions of the full-length or truncated Chromator constructs, Chro-FL (1–926), Chro-NTD (1–346), Chro-CTD (329–926), Chro-M (329600), Chro-M1 (329–460), Chro-M2 (389–531), Chro-M3 (461–600), and Chro-421 (601–926) were generated using standard methods [9] and as previously described [10]. The respective GST fusion proteins and GST only were expressed in BL21 cells and purified over a glutathione agarose column (Sigma-Aldrich) according to the pGEX manufacturer's instructions (Amersham Biosciences). For the overlay interaction assays approximate relative molar ratios of Chro-FL (10 µg), Chro-NTD (6 µg), Chro-CTD (8 µg), Chro-M (6 µg), Chro-421 (6 µg), and GST (2 µg) were fractionated by SDS-PAGE and electroblotted to nitrocellulose. The membrane was subsequently blocked in 5% non fat dry milk in TBST (TBS with 0.1% Tween-20) for 1 h, washed once in 1% non fat dry milk in TBST for 15 min, and washed once in PEMF buffer (80 mM Pipes; 2 mM MgCl_2_; 0.5 mM EGTA; 25 mM NaF) supplemented with 1 mM GTP. 5 µg/ml of purified bovine tubulin (Cytoskeleton) was polymerized with 1 mM GTP and 20 µM taxol in PEMF buffer before the blot was incubated overnight in this solution at room temperature. After being washed twice in PEMF buffer the bound microtubules were detected by standard immunoblot analysis using anti-tubulin antibody. Input proteins were analyzed by SDS-PAGE and immunoblotting with GST antibody. The cDNA sequence for all fusion proteins was verified by sequencing at the Iowa State University DNA Facility.

### Spindown assays

For in vitro spin down assays microtubules were assembled from 16 µg of commercial bovine brain tubulin monomers (Cytoskeleton) in PEM buffer and stabilized with 20 µM taxol and 2 mM GTP at 37°C for 20 min. The assembled microtubules were then incubated with approximate relative molar ratios of Chro-FL (10 µg), Chro-NTD (6 µg), Chro-CTD (8 µg), Chro-M (6 µg), Chro-421 (6 µg), and GST (2 µg) at room temperature for 30 min. Assembled microtubules and associated proteins were then pelleted by centrifugation at 75,000 rpm for 20 min. For immunoblot analysis the pellet and supernatant were carefully separated, fractionated by SDS-PAGE, immunoblotted and probed with anti-GST and anti-tubulin antibody.

For in vivo spin down assays 0–3 hour embryo protein lysates were prepared as described in Qi et al. [3] and treated with either 20 µM taxol and 2 mM GTP or with 1 mg/ml nocodazole (Sigma Aldrich). Subsequently the respective lysates were subjected to centrifugation at 75,000 rpm for 20 min. The resulting pellet and supernatant fractions were carefully separated and fractionated by SDS-PAGE, immunoblotted and probed with Chromator mAb 6H11 and anti-tubulin antibody.

For spindown binding affinity assays a constant amount of Chro-CTD (10 µg) was incubated with various concentrations (0.125–2.5 µM) of taxol-stabilized microtubules assembled from purified bovine brain tubulin as described above. Using UVP VisionWorks LS the relative bound fraction of Chro-CTD in the pellets was determined by densitometry analysis of the immunoblots. The Kd was calculated using a Lineweaver-Burk plot.

### Pull-down experiments

For in vitro pull-down assays approximate molar ratios of GST–Chromator fusion proteins or GST protein alone were coupled to glutathione agarose beads (Sigma) and incubated with 2 µg of TRITC labeled commercial bovine brain tubulin (Cytoskeleton) in 500 µl of immunoprecipitation (ip) buffer (20 mM Tris–HCl pH 8.0, 10 mM EDTA, 1 mM EGTA, 150 mM NaCl, 0.1% Triton X-100, 0.1% Nonidet P-40, 1 mM PMSF, and 1.5 µg aprotinin) overnight at 4°C. The experiments were performed either with 1 mg/ml colchicine present in the buffer to prevent tubulin polymerization or with 20 µM taxol and 2 mM GTP to generate microtubules. The protein complex coupled beads were washed five times for 10 min each with 1 ml of 2× PBS-T. After separation by SDS-PAGE and immunoblotting TRITC-tubulin was detected by epifluorescence using the UVP imaging system.

For in vivo pull-down assays of native tubulin, *Drosophila* S2 cell lysate was prepared as described in Yao et al. [6]. Approximate molar ratios of GST–Chromator fusion proteins or GST protein alone were coupled to glutathione agarose beads (Sigma) and incubated with 500 µl of S2 cell lysate at 4°C overnight. The experiments were performed either with 1 mg/ml colchicine present in the buffer to dissociate polymerized tubulin or with 20 µM taxol and 2 mM GTP to stabilize microtubules. The beads were washed five times for 10 min each in 1 ml of ip buffer, and proteins retained on the glutathione agarose beads were analyzed by SDS-PAGE and immunoblotting using anti-tubulin antibody.

### Immunoprecipitation assays

For co-immunoprecipitation experiments 5 µl of mouse anti-α-tubulin antibody, 100 µl of mAb 12H9 supernatant, or 5 µl of mAb anti-GST antibody 8C7 was bound to 30 µl protein G-Sepharose beads (Sigma) for 2.5 h at 4°C on a rotating wheel in 300 µl ip buffer. Subsequently antibody-coupled beads or beads only were incubated overnight at 4°C with 500 µl of untreated S2 cell lysate on a rotating wheel. The beads were washed three times for 10 min each with 1 ml of ip buffer with low-speed pelleting of beads between washes. The resulting bead-bound immuno-complexes were analyzed by SDS-PAGE and immunoblotting using mAb 6H11 to detect Chromator and anti-tubulin antibody to detect tubulin.

## Results

### Chromator interacts with tubulin in in vivo interaction assays

**Figure 1:**
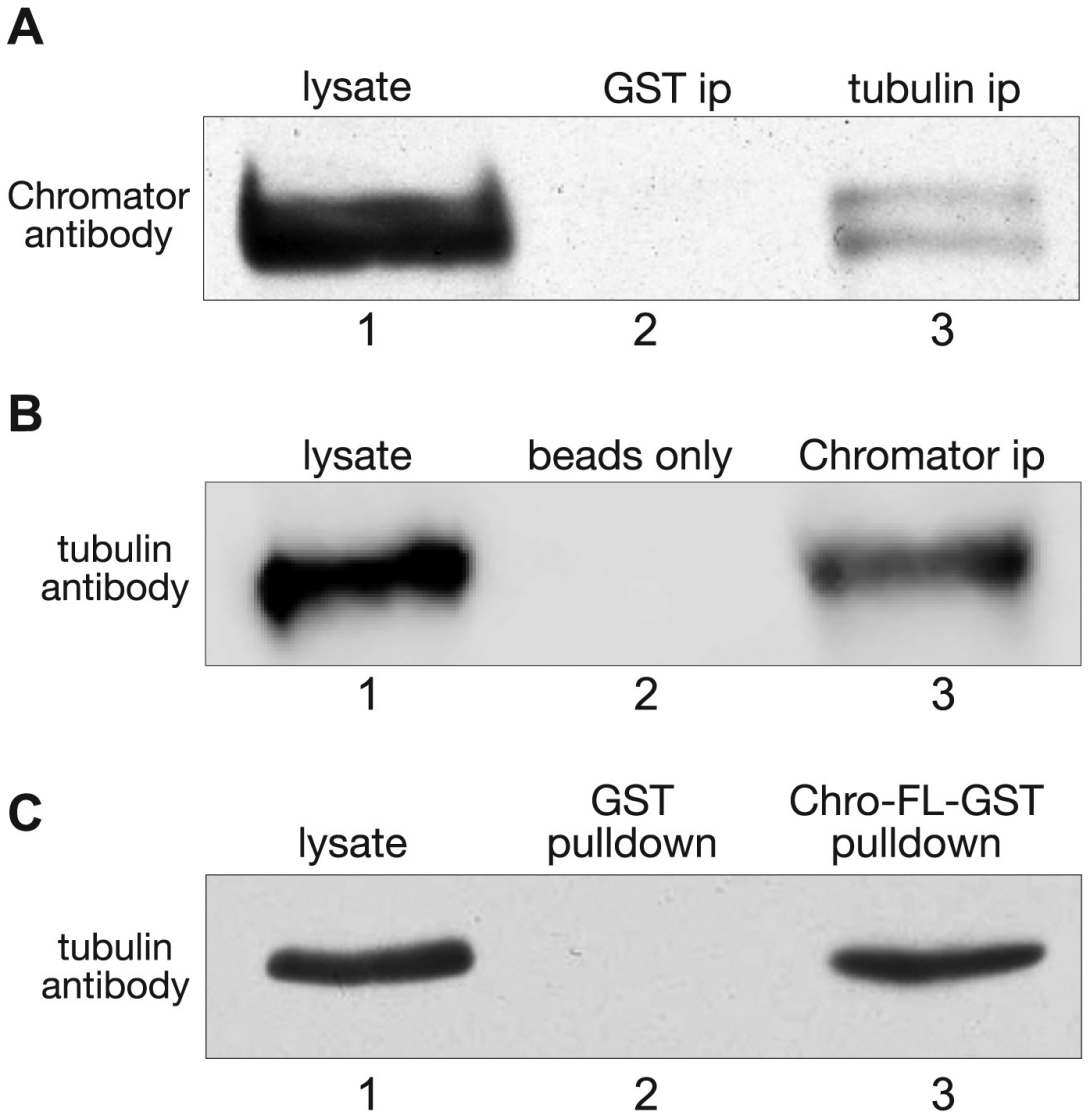
Chromator and tubulin immunoprecipitation and pulldown assays. (A) Immunoprecipitation of lysate from S2 cells using tubulin antibody and detected with Chromator antibody. Chromator is detected in the tubulin ip (lane 3) and in the S2 cell lysate (lane 1) but not in the GST antibody control ip (lane 2). (B) Immunoprecipitation of lysate from S2 cells using Chromator antibody and detected with tubulin antibody. Tubulin is detected in the Chromator ip (lane 3) and in the S2 cell lysate (lane 1) but not in the beads only control (lane 2). (C) A full-length Chromator GST-fusion construct (Chro-FL-GST) pulls down tubulin from S2 cell lysate as detected by tubulin antibody (lane 3). A GST-only pull down control was negative (lane 2). Lane 1 shows the position of tubulin in the S2 cell lysate.

In order to further probe for a potential in vivo interaction between Chromator and tubulin we performed immunoprecipitation (ip) experiments and a pull-down experiment using S2 cell lysate. For the IP experiments proteins were extracted from S2 cells, immunoprecipitated with tubulin or Chromator antibody, fractionated on SDS-PAGE after the immunoprecipitation, immunoblotted, and probed with antibody to Chromator and tubulin, respectively. Figure 1A shows an example of a tubulin antibody ip experiment labeled by Chromator antibody. Chromator was detected by the antibody both in the lysate as well as in the immunoprecipitate lanes but not in the GST ip control lane. Figure 1B shows an example of a Chromator antibody ip experiment labeled by tubulin antibody. Tubulin was detected by the antibody both in the lysate as well as in the immunoprecipitate lanes but not in the beads only control lane. Furthermore, we performed a pulldown experiment using a full-length Chromator GST-tagged construct (Chro-FL-GST). In the pull-down experiment, Chro-FL-GST was coupled to glutathione-agarose beads, incubated with S2 cell lysate, washed, fractionated by SDS-PAGE, and analyzed by immunoblot analysis using a tubulin specific antibody. A GST protein-only pull-down served as control. Whereas the GST only control showed no pull-down activity, Chro-FL-GST was able to pull-down tubulin as detected by tubulin antibody (Fig. 1C). Taken together these experiments present further evidence for an in vivo interaction between Chromator and tubulin.

**Figure 2:**
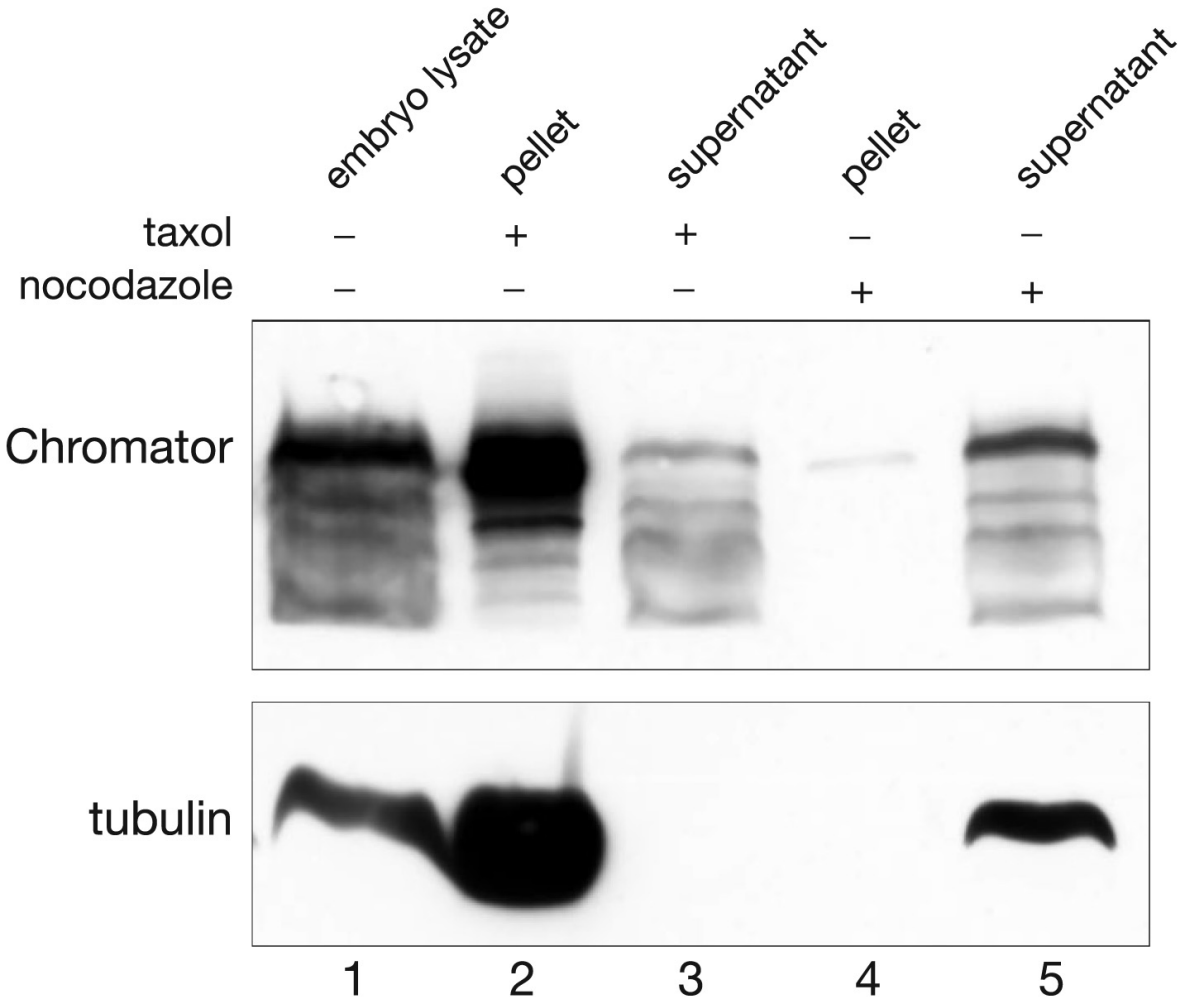
Tubulin spindown assays from 0–3 h embryonic lysates. Microtubules were either stabilized with addition of 20 µM taxol and 2 mM GTP (lane 2 and 3) or disassembled by addition of 1 mg/ml nocodazole (lane 4 and 5). Subsequently the respective lysates were subjected to centrifugation at 75,000 rpm for 20 min. The resulting pellet and supernatant fractions were separated and fractionated by SDS-PAGE, immunoblotted and probed with Chromator mAb 6H11 and anti-tubulin antibody. Lane 1 shows migration of Chromator and tubulin from untreated embryonic lysate.

To determine whether Chromator interacted with polymerized microtubules we performed spindown assays using lysate from 0–3 h *Drosophila* embryos under conditions where polymerized tubulin and associated proteins were separated into the pellet fraction and free tubulin into the supernatant fraction. In the experiments embryo lysates were treated with taxol to generate polymerized microtubules or with nocodazole to destabilize microtubules into free tubulin. Subsequently, after ultracentrifugation of the lysates the pellet and supernatant were carefully separated, fractionated by SDS-PAGE, immunoblotted, and probed with Chromator and tubulin antibody. As illustrated in Fig. 2 after taxol treatment and tubulin polymerization into microtubules the majority of both tubulin and Chromator was found in the pellet fraction (lane 2) whereas no detectable tubulin and very little Chromator was present in the supernatant (lane 3). In contrast, after nocodazole treatment and microtubule depolymerization into free tubulin the majority of both tubulin and Chromator was found in the supernatant fraction (lane 5) whereas no detectable tubulin and very little Chromator was present in the pellet (lane 4). The co-precipitation of Chromator and microtubules in these spindown assays strongly suggest that Chromator can interact with microtubules in vivo.

### A region in the carboxy-terminal domain of Chromator binds directly to microtubules

**Figure 3:**
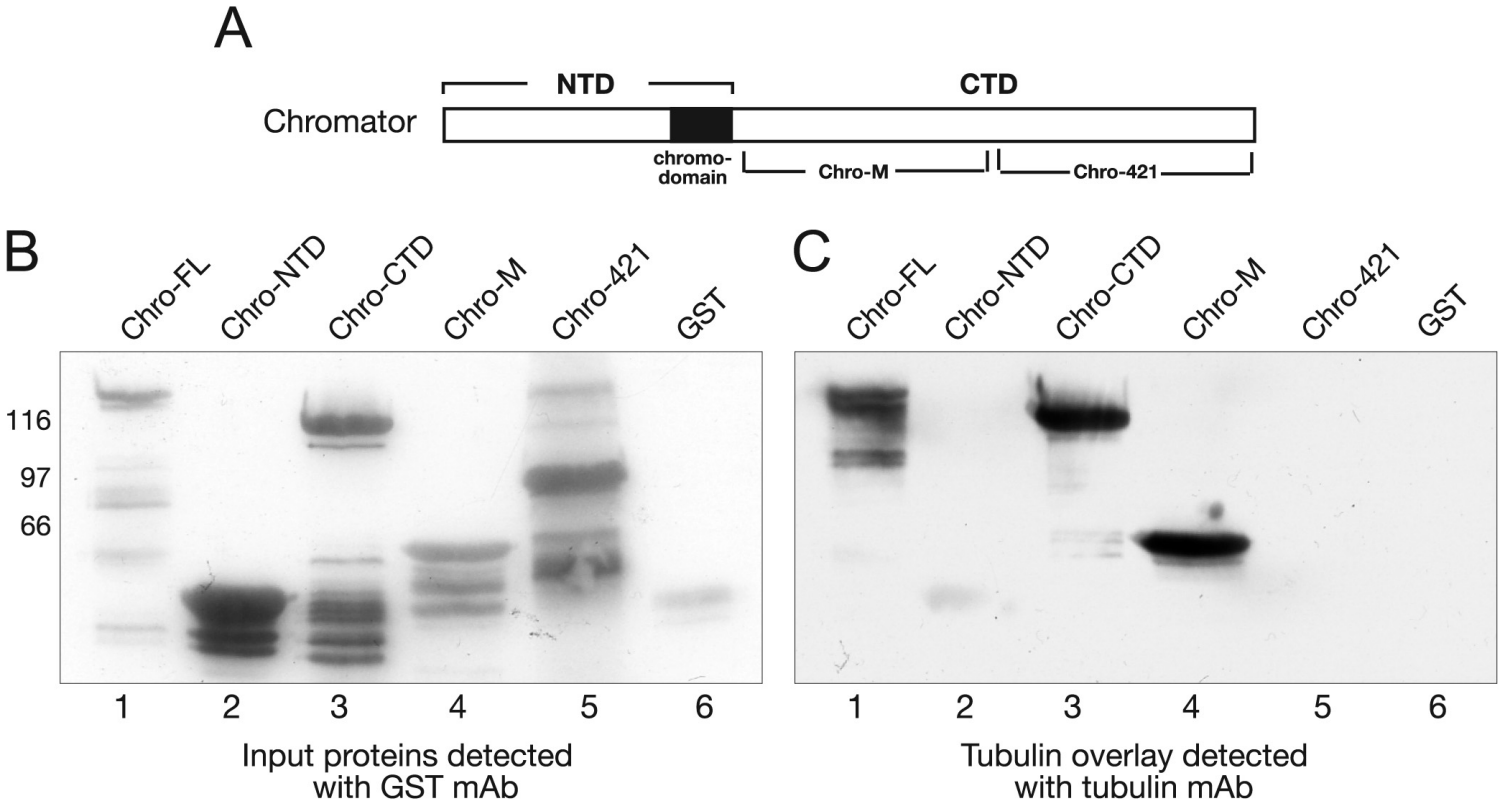
Overlay assay mapping of the Chromator interaction domain with tubulin. (A) Diagram of Chromator indicating the domains to which GST-fusion proteins were made for mapping. (B) Immunoblot of the respective GST fusion proteins and GST-only labeled with a GST mAb. (C) In the overlay experiments the Chromator GST-fusion protein constructs and GST-only shown in (B) were incubated with taxol stabilized microtubules and interactions detected with tubulin antibody. In these experiments interactions with Chro-FL, Chro-CTD, and Chro-M were detected (lane 1, 3, and 4) but not with Chro-NTD, Chro-421, and GST (lane 2, 5, and 6). This defined the Chro-M domain as sufficient for mediating interactions with tubulin. The relative migration of molecular weight markers is indicated to the left of the immunoblots in kDa.

To further characterize the interaction between Chromator and microtubules and to identify the domain mediating the interaction we performed in vitro overlay assays with polymerized tubulin of GST-fusion proteins of various regions of Chromator (Fig. 3). We used five GST-fusion proteins covering full-length (Chro-FL), the NH_2_-terminal domain (Chro-NTD), the COOH-terminal domain (Chro-CTD), and two truncated COOH-terminal domains (Chro-421 and Chro-M) as diagrammed in Fig. 3A. Figure 3C shows Chromator GST-fusion proteins that were fractionated by SDS-PAGE, transferred to nitrocellulose paper, and incubated with 5 µg/ml of tubulin polymerized with 20 µM taxol and 1 mM GTP. Protein interactions were detected with tubulin antibody. As illustrated in Fig. 3C Chro-FL, Chro-CTD as well as Chro-M were found to interact with tubulin in these assays but not Chro-NTD, Chro-421, or the GST control. Immunoblot analysis of the GST proteins purified in these experiments and detected with GST-antibody showed that similar levels of the GST-fusion proteins were present in the overlay assay (Fig. 3B). Thus, these results indicate that Chromator sequences in the Chro-M domain can directly bind to microtubules.

**Figure 4:**
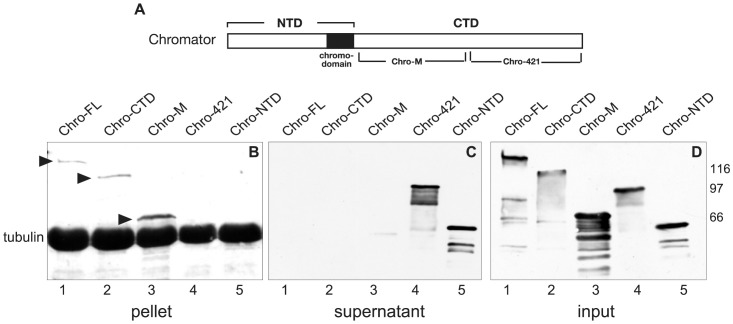
Spindown assay mapping of the Chromator interaction domain with tubulin. (A) Diagram of Chromator indicating the domains to which GST-fusion proteins were made for mapping. (B–C) In the spindown experiments the Chromator GST-fusion protein constructs were incubated with taxol stabilized microtubules. Assembled microtubules and associated proteins were then pelleted by centrifugation at 75,000 rpm for 20 min. For immunoblot analysis the pellet (B) and supernatant (C) were separated, fractionated by SDS-PAGE, immunoblotted and probed with anti-GST and anti-tubulin antibody. In these experiments Chro-FL, Chro-CTD, and Chro-M were detected in the pellet fraction (B) whereas Chro-NTD and Chro-421 were detected in the supernatant (C). This confirmed the Chro-M domain as sufficient for mediating interactions with tubulin. Tubulin for all five experimental conditions were only detectable in the pellet (B). (D) Immunoblot of the respective GST fusion proteins used in the spindown assays labeled with a GST mAb. The relative migration of molecular weight markers is indicated to the right of the immunoblots in kDa.

In order to confirm the above results we also performed in vitro spindown assays. In these experiments microtubules were assembled from bovine tubulin monomers with 20 µM taxol and 1 mM GTP and incubated with the different Chromator GST-fusion proteins (Fig. 4). Subsequently, after ultracentrifugation of the samples the pellet and supernatant were carefully separated, fractionated by SDS-PAGE, immunoblotted, and probed with Chromator and tubulin antibody. As illustrated in Fig. 4B all three Chromator GST-fusion proteins containing the M-domain, Chro-FL, Chro-CTD, and Chro-M were found in the pellet fraction but not in the supernatant. In contrast, Chro-421 and Chro-NTD were largely present in the supernatant (Fig. 4C). Furthermore, almost all the tubulin for all five experimental conditions were present in the pellet. Immunoblot analysis of each of the input GST fusion proteins probed with anti-GST antibody showed comparable levels of GST fusion proteins in each of the spindown assays (Fig. 4D). Thus, the findings from the spindown assays were identical to those of the overlay assays further confirming a direct interaction of Chromator's M-domain with microtubules.

**Figure 5:**
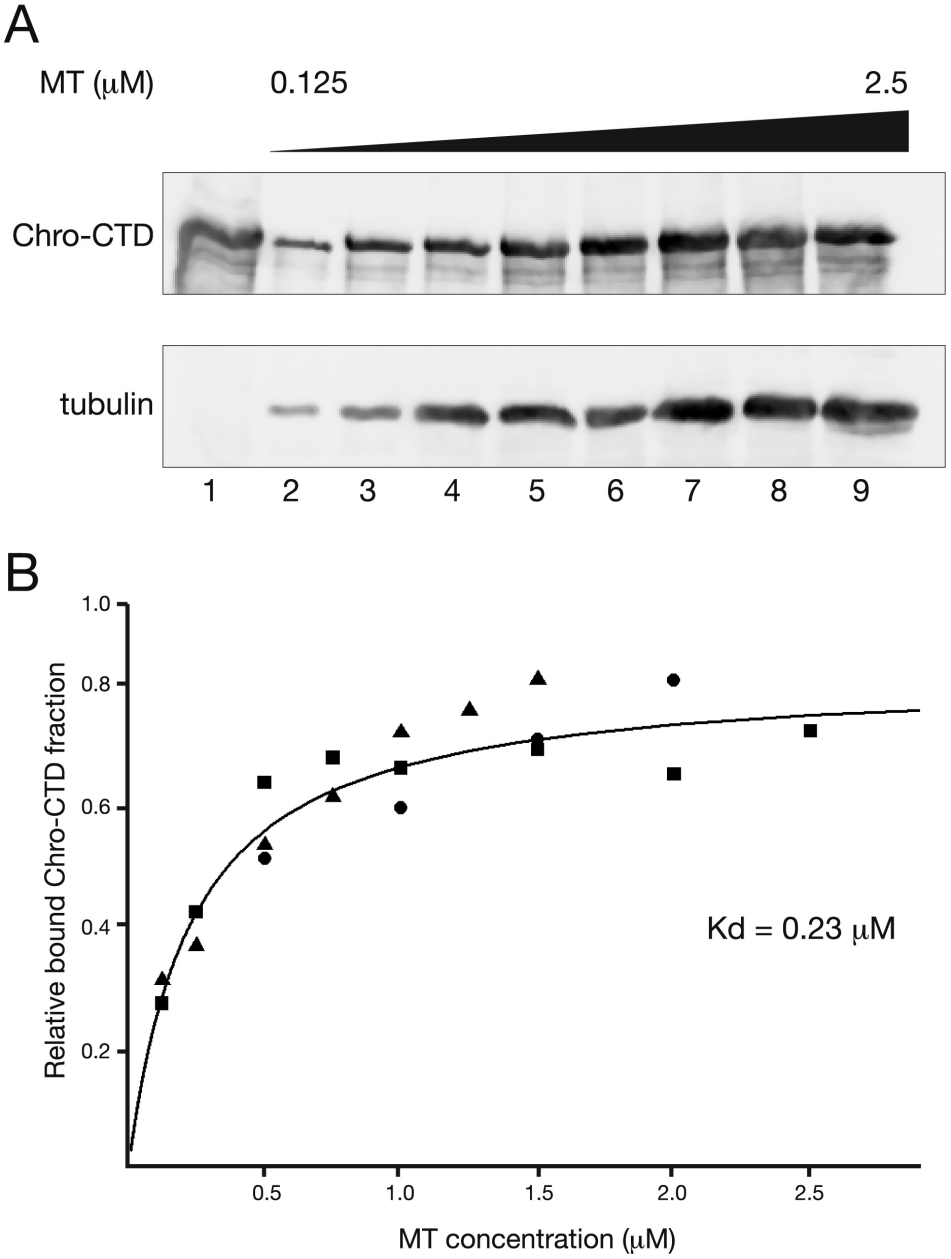
Binding affinity of Chromator-CTD for microtubules determined by spindown assays. (A) Immunoblot analysis of spindown assays of Chro-CTD binding to various concentrations of taxol-stabilized microtubules (MT). 10 µg of Chro-CTD (lane 1) was incubated with microtubules at concentrations ranging from 0.125–2.5 µM (lane 2–9). Chro-CTD was detected with mAb 6H11 and microtubules with tubulin antibody. (B) Binding curve for Chro-CTD with microtubules based on three independent experiments indicated by squares, circles, and triangles, respectively. The calculated Kd was 0.23 µM.

In order to determine the binding affinity of Chromator for microtubules we incubated various concentrations of microtubules assembled from bovine tubulin monomers with 20 µM taxol and 1 mM GTP with a constant amount of Chro-CTD (10 µg) and performed spindown assays as described above. As illustrated in Fig. 5A analysis by SDS-PAGE and immunoblotting showed increasing amounts of Chro-CTD in the pellets with increasing microtubule concentration. We determined the bound Chro-CTD protein fractions from three independent experiments and plotted them against microtubule concentration which gave a binding curve (Fig. 5B) with a Kd of 0.23 µM which is very similar to that of 0.25 µM determined for the *Drosophila* microtubule binding protein, Mars [11].

### Chromator directly interacts with unpolymerized free tubulin

**Figure 6:**
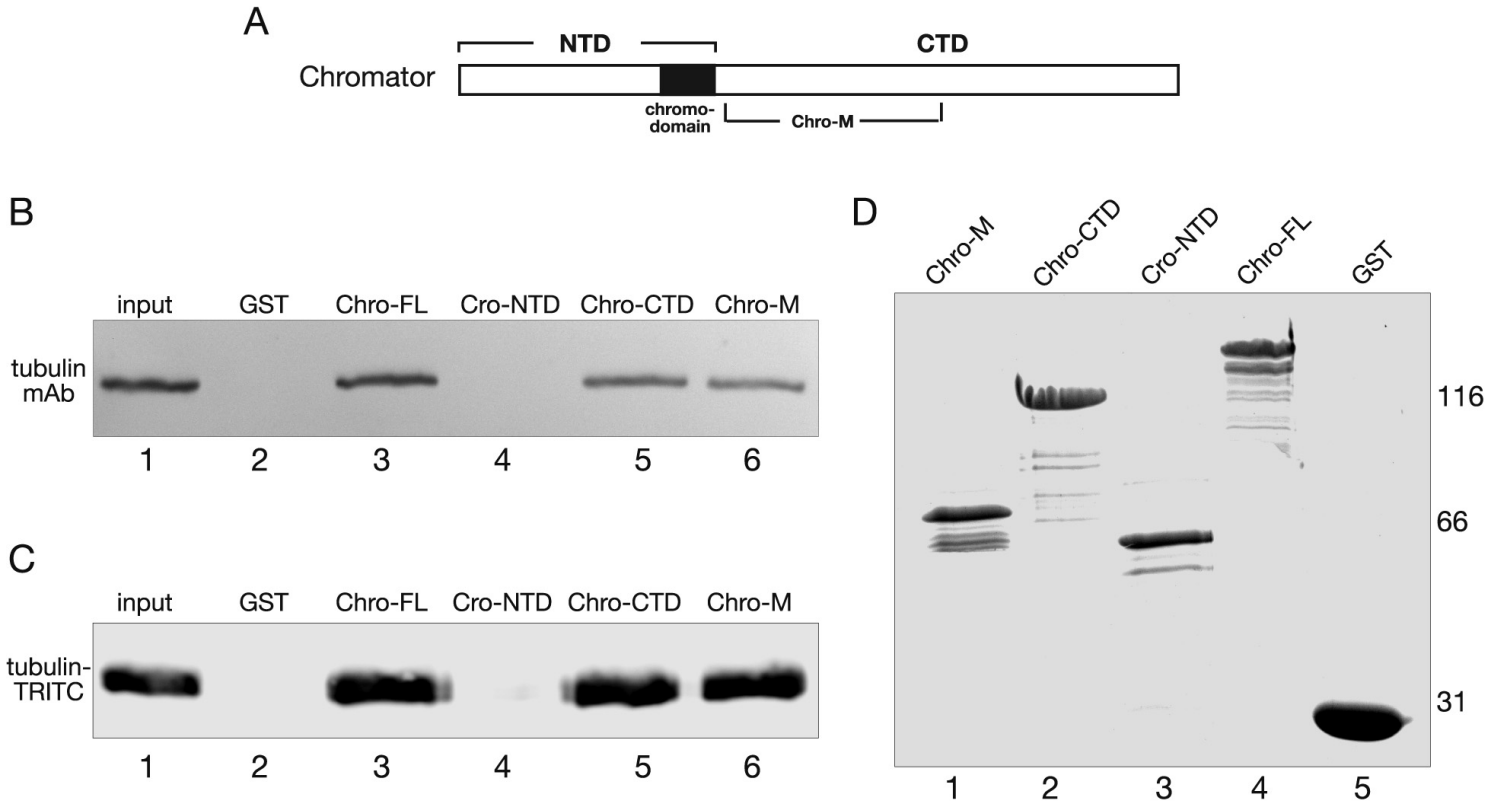
Chromator directly interacts with unpolymerized free tubulin. (A) Diagram of Chromator indicating the domains to which GST-fusion proteins were made for mapping. (B) Pulldown assays from S2 cell lysate incubated with Chromator-GST fusion proteins or GST only under conditions where microtubules were depolymerized by colchicine. Bound proteins were washed, fractionated by SDS-PAGE, immunoblotted, and analyzed using a tubulin specific antibody. Whereas Chro-NTD and the GST only control showed no pull-down activity (lane 2 and 4), Chro-FL, Chro-CTD and Chro-M were all able to pull-down tubulin (lane 3, 5, and 6) as detected by tubulin antibody. Lane 1 shows tubulin from untreated S2 cell lysate. (C) Pulldown assays with bovine TRITC-labeled tubulin incubated with Chromator-GST fusion proteins or GST only under conditions where microtubules were prevented from forming by colchicine. Bound proteins were washed, fractionated by SDS-PAGE, immunoblotted, and analyzed for TRITC fluorescence. Whereas Chro-NTD and the GST only control showed no pull-down activity (lane 2 and 4), Chro-FL, Chro-CTD and Chro-M were all able to pull-down tubulin (lane 3, 5, and 6). Lane 1 shows TRITC-tubulin. These experiments defined the Chro-M domain as sufficient for mediating interactions with unpolymerized free tubulin. (D) Immunoblot of the respective GST fusion proteins and GST used in the pulldown assays labeled with a GST mAb. The relative migration of molecular weight markers is indicated to the right of the immunoblots in kDa.

Yao et al. [5] recently presented evidence by injection of high molecular weight dextrans into syncytial embryos that the disassembling nuclear envelope and nuclear lamina after their initial breakdown, are not likely to present a diffusion barrier to most known proteins during mitosis. Interestingly, even in the absence of such a diffusion barrier free tubulin (possibly as α/β-tubulin dimers) accumulates co-extensively with Chromator in colchicine-treated embryos independently of tubulin polymerization [5]. The level of unpolymerized tubulin enrichment within the Chromator defined matrix in the nuclear space was about 1.6 fold the levels outside the nuclear space [5]. Thus, in order to determine whether Chromator has the capacity to interact with free tubulin in addition to microtubules we performed pulldown assays from S2 cell lysate with Chromator-GST fusion proteins under conditions where microtubules were depolymerized by colchicine. In the experiments the different Chromator GST-fusion constructs (Fig. 6A) were coupled to glutathione beads and incubated with 1 mg/ml colchicine treated S2 cell lysate. Bound proteins were washed, fractionated by SDS-PAGE, immunoblotted, and analyzed using a tubulin specific antibody. A GST protein only pull-down served as a control. Whereas Chro-NTD and the GST only control showed no pull-down activity, Chro-FL, Chro-CTD and Chro-M were all able to pull-down tubulin as detected by tubulin antibody (Fig. 6B). In order to confirm these results we applied the same experimental paradigm except for substituting purified bovine TRITC-labeled tubulin for the S2 cell lysate. Bound proteins were washed, fractioned by SDS-PAGE, blotted, and the blots analyzed for TRITC immunofluorescence. As illustrated in Fig. 6C an identical result to that for tubulin pulldown from S2 cell lysate was obtained. Chro-NTD and the GST only control showed no pull-down activity, whereas Chro-FL, Chro-CTD and Chro-M were all able to pull-down tubulin (Fig. 6C). Gel analysis of each of the input GST fusion proteins labeled with coomassie blue showed comparable levels of GST fusion proteins in each of the pulldown assays (Fig. 6D). Taken together these experiments indicate that the M-domain of Chromator has the capacity to bind to unpolymerized free tubulin as well as to microtubules.

### A Chro-M subdomain interacts with microtubules but not with free tubulin

**Figure 7:**
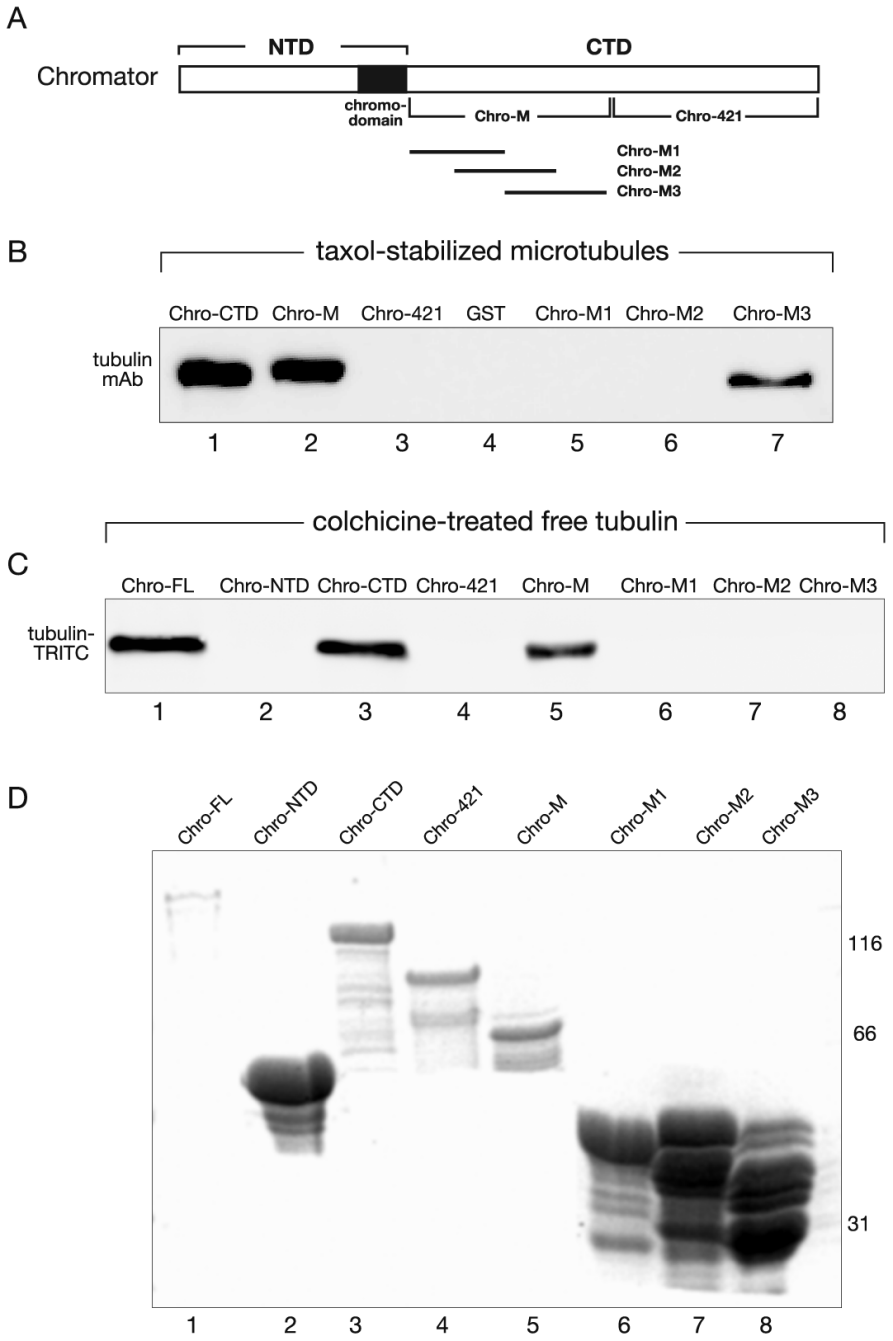
A Chro-M subdomain is minimally required for polymerized tubulin binding activity. (A) Diagram of Chromator indicating the domains to which GST-fusion proteins were made for mapping including the overlapping set of Chro-M1-M3 spanning the Chro-M domain. (B) Pulldown assays with purified bovine tubulin incubated with Chromator-GST fusion proteins under conditions where microtubules were assembled and stabilized by taxol. Bound proteins were washed, fractionated by SDS-PAGE, immunoblotted, and analyzed using a tubulin specific antibody. Whereas Chro-421, GST and Chro-M1-M2 showed no pull-down activity (lane 3–6), Chro-CTD, Chro-M, and Chro-M3 were all able to pull-down tubulin (lane 1, 2, and 7). (C) Pulldown assays with bovine TRITC-labeled tubulin incubated with Chromator-GST fusion proteins under conditions where microtubules were prevented from forming by colchicine. Bound proteins were washed, fractionated by SDS-PAGE, immunoblotted, and analyzed for TRITC fluorescence. Whereas Chro-NTD, Chro-421, and Chro-M1-M3 showed no pull-down activity (lane 2, 4, 6, 7, and 8), Chro-FL, Chro-CTD and Chro-M were all able to pull-down tubulin (lane 1, 3, and 5). (D) Immunoblot of the respective GST fusion proteins used in the pulldown assays labeled with a GST mAb. The relative migration of molecular weight markers is indicated to the right of the immunoblots in kDa.

In an attempt to further define the minimally required amino acid sequence for Chromator's tubulin binding activity we made an overlapping set of three GST-fusion proteins, Chro-M1-M3, spanning the Chro-M domain as diagrammed in Fig. 7A and performed pull-down experiments of both polymerized and free tubulin as described above. Interestingly, only microtubules (Fig. 7B) but not free tubulin could be pulled down by Chro-M3 (Fig. 7C) whereas Chro-M1 and Chro-M2 had no detectable pulldown activity. As in the previous experiments the Chro-FL, Chro-CTD, and Chro-M constructs showed strong pull-down activity (Figs. 7B and 7C). These results indicate that residues 461–600 within the Chro-M3 domain are sufficient for microtubule binding activity whereas additional residues within the Chro-M domain are necessary for binding of free tubulin. Data base searches of the Chro-M3 sequence did not reveal any known microtubule binding motifs suggesting that this sequence defines a novel tubulin binding interphase.

## Discussion

The concept of a spindle matrix has long been proposed [12], [13]; however, whether such a structure exists and its molecular composition and how it may interact with the microtubule-based spindle apparatus has remained controversial (reviewed in [14]–[19]). In this study using a variety of biochemical assays we show that the spindle matrix protein, Chromator, can directly interact with microtubules as well as with free tubulin. Furthermore, we have mapped this interaction with tubulin to a relatively small stretch of 271 aa in the carboxy-terminal region of Chromator. This sequence is likely to contain a novel tubulin binding interface since database searches did not find any sequence matches with known tubulin binding motifs.

The microtubule-based spindle apparatus provides a conserved mechanism to segregate chromosomes during mitosis. However, how this process is coordinated with disassembly and reassembly of nuclear structures during mitotic progression is poorly understood [20]. It is also not clear how cell cycle regulators and other diffusible molecules are localized and confined to the spindle region in the absence of diffusion barriers following nuclear envelope breakdown [16], [18], [21]. To begin to address these issues Yao et al. [5] depolymerized tubulin by injecting colchicine into syncytial embryos prior to prophase. Under these conditions Chromator still relocated from the chromosomes to the matrix; however, in the absence of microtubule spindle formation the Chromator-defined matrix did not undergo any dynamic changes but instead statically embedded the condensed chromosomes for extended periods. Moreover, unpolymerized tubulin accumulated within the nuclear space relative to the levels outside the nuclear space in the colchicine injected embryos. A similar enrichment within the nuclear region of free tubulin after nuclear envelope breakdown has been reported in *C. elegans* embryos [22]. Thus, the enhanced accumulation of free tubulin within the nascent spindle region may serve as a general mechanism to promote the efficient assembly of the microtubule-based spindle apparatus [22] and be mediated by spindle matrix constituents. Based on Chromator's ability to bind free tubulin we propose that Chromator may fulfill such a role in *Drosophila*.

The mechanisms and relative dynamics of Chromator's interaction with free tubulin and microtubules are not known. However, recent studies of membrane-less macromolar assemblies, such as ribonucleoprotein granules/bodies (RNP droplets), P granules, Cajal bodies, nucleoli, and the centrosome, which may have a structure similar to the spindle matrix, indicate that they are highly dynamic (reviewed in [23]). Weak, repetitive interactions between the macromolecules making up these assemblies facilitate the formation of a coherent structure in the absence of a membrane, while still enabling a fluid-like micro-environment similar to that of membrane-bound organelles [23]. Studies have indicated that these structures can function as liquid phase micro-reactors, concentrating various protein components and accelerating the kinetics of protein-protein reactions (reviewed in [23]). Chromator interactions with free tubulin within the context of the spindle matrix would be consistent with such a scenario.

Moreover, it has recently been demonstrated that Megator and its human homolog Tpr act as spindle matrix proteins that have an evolutionarily conserved function as spatial regulators of the spindle assembly checkpoint that ensure the efficient recruitment of Mad2 and Mps1 to unattached kinetochores in eukaryotes from fungi to humans during mitosis [20], [24]–[26]. Taken together with the present demonstration of Chromator's tubulin binding activity these findings provide support for the hypothesis that reorganization of nuclear proteins into a spindle matrix may play a wider functional role in spatially regulating cell cycle progression factors in conjunction with contributing to microtubule spindle assembly and dynamics. Thus, future studies of Chromator and other spindle matrix proteins are likely to provide new insights into how cell cycle factors are physically confined and organized in the spindle region in organisms with open or semi-open mitosis allowing for spatial and temporal control of mitotic progression and chromosome segregation.
